# A reductionist approach to dissecting grain weight and yield in wheat

**DOI:** 10.1111/jipb.12741

**Published:** 2019-01-15

**Authors:** Jemima Brinton, Cristobal Uauy

**Affiliations:** ^1^ John Innes Centre Norwich Research Park Norwich NR4 7UH United Kingdom

## Abstract

Grain yield is a highly polygenic trait that is influenced by the environment and integrates events throughout the life cycle of a plant. In wheat, the major grain yield components often present compensatory effects among them, which alongside the polyploid nature of wheat, makes their genetic and physiological study challenging. We propose a reductionist and systematic approach as an initial step to understand the gene networks regulating each individual yield component. Here, we focus on grain weight and discuss the importance of examining individual sub‐components, not only to help in their genetic dissection, but also to inform our mechanistic understanding of how they interrelate. This knowledge should allow the development of novel combinations, across homoeologs and between complementary modes of action, thereby advancing towards a more integrated strategy for yield improvement. We argue that this will break barriers in terms of phenotypic variation, enhance our understanding of the physiology of yield, and potentially deliver improved on‐farm yield.

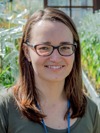

Jemima Brinton

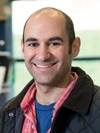

Cristobal Uauy

**Edited by:** Thorsten Schnurbusch, Leibniz Institute of Plant Genetics and Crop Plant Research (IPK), Germany

## INTRODUCTION

There is consensus that urgent action is needed to sustainably increase global food production (Godfray et al. 2010). Among the major crops, wheat (*Triticum aestivum*) accounts for over 20% of the calorific intake of humans and provides more protein (∼23%) than all animal sources combined (FAO 2017). Increases in wheat yield could have a major impact on global food and nutritional security. However, current trends will be insufficient to meet future demands (Ray et al. 2013), and this could be exacerbated by changing weather patterns (Asseng et al. 2016).

Final grain yield is the ultimate result of plant growth and therefore most, if not all, genes will contribute towards yield either directly or indirectly. As a result, achieving increased yield is a non‐trivial task, and the cumulative knowledge from wheat breeding suggests we will require simultaneous improvements of both the ‘source’ and ‘sink’ tissues. Several strategies can be used to achieve these improvements, although there are fundamental differences in the rationale behind these approaches (Reynolds et al. 2009; Foulkes et al. 2011; Parry et al. 2011).

Some strategies are based on a reductionist approach, where one seeks to describe final grain yield by studying and understanding the individual constituent parts (e.g. spikes/m^2^, grain number/spike, and grain weight). Alternatively, the complexity of the biological system is such that an integrated approach is warranted, where final grain yield needs to be studied within the framework of a whole system, rather than by the study of the individual components.

Here, we argue that a reductionist approach is required as an entry point to understand the gene networks and mechanisms regulating individual sub‐components underlying yield. This will require dissection beyond the traditional yield components (spikes/m^2^, grain number/spike, and grain weight), which are themselves highly complex and polygenic traits. Importantly, this reductionist approach should not be confused with studying sub‐components in isolation. We suggest that, by understanding the precise mechanisms by which individual genes regulate individual yield components, we will be better positioned to understand, and potentially decouple, negative correlations between yield components.

The mechanistic knowledge acquired can then be used to assemble allele combinations in an informed and targeted manner. These combinations will effectively exploit the polyploid context present in wheat and optimize individual, or multiple yield components across different environments, which can then be tested under local farm management practices. This is becoming increasingly possible due to the availability of novel genomic sequences and resources (Borrill et al. 2016; Zhang et al. 2016b; Krasileva et al. 2017; Mascher et al. 2017; IWGSC 2018) and our knowledge of pathways from model species and wheat itself (Li and Li 2015; Li and Yang 2017).

Here we focus on grain weight, an important yield component, which is stably inherited (Kuchel et al. 2007) and is, itself, comprised of multiple sub‐components, including carpel size, grain morphometric parameters (length, width, height), and the rate of grain filling. We provide a general overview of the multiple processes occurring during grain development, both across the spike as well as within an individual grain. We then discuss the importance of focusing on specific grain weight sub‐components, not only to help in the genetic dissection of grain weight, but also to inform our mechanistic understanding of how they operate and interact with other factors. We argue that this knowledge is essential to modulate grain weight and additional yield components to ensure they deliver improved on‐farm yield. Finally, we discuss some of the future challenges and opportunities.

Throughout the review, we focus primarily on studies from wheat, but also refer to insights from other cereals and model species, including barley (*Hordeum vulgare*), rice (*Oryza sativa*) and *Arabidopsis thaliana*. It is important to note that there are fundamental differences between the cereal grain (e.g. wheat, barley, rice) and the seeds of other model species, such as *Arabidopsis*. All seeds are surrounded by a layer of cells, known as the seed coat, which is derived from the maternal integuments. In the case of cereals, however, the grain is a fruit rather than a true seed. This fruit, called a caryopsis, has its seed coat fused to the pericarp, another tissue of maternal origin, which is derived from the ovary wall. The cereal caryopsis is a dry, single seeded fruit, formed from a single carpel and is indehiscent, meaning that it does not open and release the true seed at maturity. The *Arabidopsis* seed, on the other hand, is a true seed contained within a dehiscent fruit, which in turn is formed from two carpels and houses many individual seeds.

Therefore, while some processes and mechanisms will be conserved across species, there will also be differences in the ways in which grain and seed development are regulated (for more detailed comparisons see Linkies et al. 2010; Taiz and Zeiger 2014). For the purpose of this review, we will use the term ‘grain’ to refer explicitly to the cereal caryopsis and ‘seed’ to refer to the *Arabidopsis* seed. When it is necessary to refer to the two, simultaneously, we will use the term ‘seed’.

## GRAIN WEIGHT ACROSS A SPIKE

**Figure 1:**
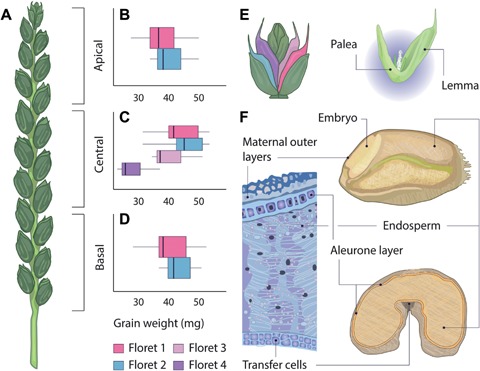
**Grain weight across a wheat inflorescence (spike) and structure of mature grain** The wheat inflorescence, known as the spike (**A**) consists of multiple spikelets, each comprising two outer glumes and multiple florets (**E**) that have the potential to hold grain. Each floret has two sheathing structures, the lemma and palea, which enclose the carpel and three stamens. Grain weight is non‐uniform across the spike, with spikelets in the apical (**B**) and basal (**D**) sections of the spike having smaller grains than the central spikelets (**C**). Grain weight is also non‐uniform within a single spikelet. At all spikelet positions, floret 2 contains the largest grain and the size of the grain from floret 1 is similar, but slightly smaller (**B–D**). Grains produced from floret 3 and 4 are smaller than those in florets 1 and 2 (**C**). **F** illustrates longitudinal (upper) and cross (lower) sections of a mature wheat grain, with a representation of the cell types found in a cross section (left). The aleurone, transfer cells and starchy endosperm are differentiated endosperm cell types and the maternal layers shown consist of the outer pericarp, cross cells and seed coat (top to bottom). Data for panels **B**–**D** from (Calderini and Ortiz‐Monasterio 2003; Liu et al. 2006; Lizana et al. 2010).

Each wheat plant will produce multiple inflorescences, commonly referred to as spikes, and each spike is comprised of flowers arranged in specialized branches, termed spikelets, which are attached to a main axis (rachis), on opposite sides, and in an alternating pattern. A typical wheat spike will have between 15 to 20 spikelets and each spikelet is comprised of two outer glumes and between four to six fertile florets that have the potential to hold grain (Figure 1; Kirby and Appleyard 1987). Each floret in turn has two sheathing structures, the lemma and palea, which envelop the carpel and three stamens (Kirby and Appleyard 1987). It is within this floral structure, surrounded by the lemma and palea, that the carpel will be fertilized and a grain will develop.

Final grain weight is influenced by many factors, both genetic and environmental. Indeed, there is a high level of variation in grain weight within a single genotype, and even across a single wheat spike. Not all spikes and spikelets initiate and develop at the same time, and there can be several days or weeks between the initiation of the first and last spikelet. However, the primordia grow and develop at different rates, meaning that anthesis (flowering) will occur within a few days across a single spike. Within a single spike, spikelet differentiation and development begins in the middle of the spike and continues, bi‐directionally, towards the top and bottom (Bonnett 1936; Kirby 1974). As a result, several studies have shown that grain weight is higher in the central spikelets compared to the apical (top) and basal (bottom) spikelets (Figure 1B–D; Calderini and Ortiz‐Monasterio 2003; Liu et al. 2006).

Floret development within a single spikelet also occurs sequentially, but in a uni‐directional manner, starting at the bottom with floret 1 and proceeding upwards on alternating sides of the spikelet meristem. In general, the later initiated florets produce smaller grains. The initiation of the two most basal florets (floret 1 and 2) occurs at roughly the same time resulting in grains of similar size, however, the largest grain is consistently produced by floret 2 (Figure 1B–D; Calderini and Ortiz‐Monasterio 2003; Liu et al. 2006; Lizana et al. 2010; Xie et al. 2015).

Each spikelet will initiate between 8 and 12 floret primordia, although usually just the first four to six florets are potentially fertile and produce a carpel, consisting of the ovary, style and stigma (Kirby and Appleyard 1987; Evers and Millar 2002). A strong association has been observed between carpel size and final grain weight, suggesting that grain weight can be determined, maternally, even before grain development *per se* has started (Calderini et al. 1999; Hasan et al. 2011; Guo et al. 2015; Reale et al. 2017).

## OVERVIEW OF GRAIN DEVELOPMENT

**Figure 2:**
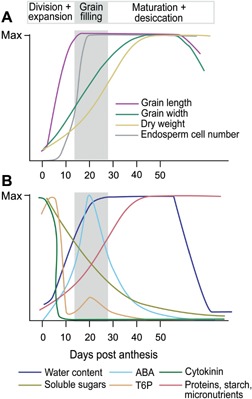
**Dynamics of grain dimensions and contents during wheat grain development** Schematic summarizing the changes in wheat grain weight and dimensions (**A**) and grain contents (**B**) across development, expressed as days post‐anthesis (dpa). Curves show the level of each parameter relative to the maximum level at any point during grain development. Shading indicates the phases of grain development: Division + expansion (0–14 dpa), Grain filling (14–28 d) and Maturation + desiccation (28 dpa onwards; from Shewry et al. 2012). ABA = abscisic acid, T6P = trehalose 6‐phosphate. Data summarized from (Sofield et al. 1977; Rogers and Quatrano 1983; Dominguez and Cejudo 1996; Hess et al. 2002; Nadaud et al. 2010; Martínez et al. 2011; Shewry et al. 2012; Xie et al. 2015).

Grain development in wheat and barley begins with the fertilization of the ovary, a maternal structure containing the ovule, and ends with the mature grain composed of three main tissues; the embryo, endosperm and maternal outer layers (Figure 1F). These tissues contain large amounts of starch, proteins and other nutrients which are accumulated during grain development. We discuss below the processes underlying grain development and we summarize their dynamics in Figure 2.

These data originate from multiple independent studies that mostly report these values as days post anthesis (dpa). This makes direct comparisons difficult due to genetic differences between cultivars tested and varying environmental conditions (e.g. temperature) which affects the rate of these processes. Our aim here is to provide a schematic overview of the general relationships among these processes and, therefore, we have combined the data into Figure 2. However, this argues for the need for a more standardized reporting of data related to grain development, for example using thermal time after anthesis in degree days (Xie et al. 2015).

The ovule in both wheat and barley comprises the haploid embryo sac, surrounded by diploid nucellus tissue and two integuments (Wilkinson et al. 2018). Upon pollen release, also referred to as anthesis, a “double fertilization” event takes place in the embryo sac, during which the triploid endosperm nucleus (a single pollen nucleus fused with two polar nuclei in the embryo sac) and the diploid embryo (the second pollen nucleus fused with the egg nucleus) are formed. These structures are enclosed by several layers of maternal tissue, including the seed coat, derived from the integuments and the pericarp, which originates from the ovary wall (Olsen 2001; Shewry et al. 2012).

In the first few dpa, the grain increases in size relative to the ovary, but the overall shape remains similar (“a blunt inverted cone”; Drea et al. 2005). During this time, the endosperm nuclei undergo several rounds of mitosis, in the absence of cytokinesis or cell wall formation. This process gives rise to the endosperm coenocyte, a multinucleate cell with a large vacuole (Olsen 2001; Drea et al. 2005). At this time, cells in the outer layers of the grain are actively proliferating (Drea et al. 2005). Cytokinin levels in the grain are at their highest during this early stage (0–7 dpa) and may act to initiate the period of rapid cell division (Hess et al. 2002). Likewise, the sugar signal metabolite trehalose‐6‐phosphate (T6P), which regulates growth and development in response to assimilate availability, is at its highest in both maternal and filial tissues at these early stages of grain development and pre‐grain filling (Martínez et al. 2011; O'Hara et al. 2013).

After an initial period of isotropic growth, the length of the developing grain increases significantly, reaching maximum length at around 15 dpa (Rogers and Quatrano 1983; Xie et al. 2015; Brinton et al. 2017). Cellularization of the endosperm begins by 6 dpa and from this point onwards the endosperm expands rapidly due to both cell division and expansion. Conversely, cell proliferation in the outer layers of the grain has declined by 6 dpa and, subsequent, growth in these tissues is mainly due to cell expansion (Drea et al. 2005; Radchuk et al. 2011).

Concurrent with endosperm cellularization, the endosperm also undergoes differentiation into the main cell types: starchy endosperm, aleurone and transfer cells (Figure 1F). The presence of multiple different cell types is one major difference between the endosperm of cereal grains and some dicots, including *Arabidopsis*, which only retain one major cell type in the endosperm of mature seeds (reviewed by Olsen 2001).

After the basic structure of the grain has been established, the grain filling period begins. This involves the accumulation of multiple storage components, including starch and proteins, such as gliadins and glutenins, in addition to micronutrients, such as iron, zinc and calcium (Sofield et al. 1977; Mecham et al. 1981; Johansson et al. 1994; Dominguez and Cejudo 1996; Gupta et al. 1996; Shewry et al. 2009). While the grain filling rate is at its highest (∼14–28 dpa; Shewry et al. 2012) the dry weight of the grain approximately doubles, and the grain volume continues to increase, but not in the longitudinal direction (Yang et al. 2006; Shewry et al. 2012; Xie et al. 2015).

Abscisic acid (ABA) content of the grain is positively correlated with grain filling rate, peaking while the grain filling rate is maximal and may be important for nutrient remobilization and grain filling processes, such as starch accumulation (Yang et al. 2006; Seiler et al. 2011). The water content of the grain also reaches its maximum during this time and is maintained until the grain reaches a maximum dry weight at around 40 dpa. Shortly after the grain has reached maximum dry weight and volume, i.e., physiological maturity, desiccation and maturation of the grain begins. This is characterized by rapid water loss from the grain, resulting in a decrease in overall grain volume, attributable to slight reductions in all grain size parameters (Xie et al. 2015).

In addition to cell division and expansion, programmed cell death (PCD) also plays an important role throughout grain development, occurring in various tissues at different stages. Shortly after fertilization, most of the nucellus undergoes PCD and the remaining tissue differentiates into the nucellar projection, a tissue critical for the delivery of nutrients from the mother plant to the endosperm, via the transfer cells (Figure 1F; reviewed in Radchuk and Borisjuk 2014). PCD also occurs in the pericarp from as early as 4 dpa when the tissue is formed of many different layers (Radchuk et al. 2011). By approximately 15 dpa, the pericarp consists of fewer layers and is significantly reduced in thickness due to PCD. Conversely, PCD in the endosperm takes place slightly later, occurring in certain areas from 16 dpa and across the whole endosperm by 30 dpa (Young and Gallie 1999). Some tissues in the grain remain alive at maturity, such as the aleurone layer and embryo (reviewed in Domínguez and Cejudo 2014).

## GENETIC CONTROL OF GRAIN WEIGHT

While most phases of grain development have been extensively characterized phenotypically, the genetic basis of how these processes are controlled and how they influence final grain weight is not well understood in wheat. Transcriptomic and proteomic studies have provided a global overview of the types of genes that are involved in grain development. These remain largely descriptive, however, and often focus on the middle and later stages of grain filling, despite the importance of early events during carpel and grain development. For example, genes enriched for ontologies, including cell division, photosynthesis, nucleic acid and protein metabolism, are preferentially expressed at the very early stages of grain development, consistent with the ongoing cellular processes (Laudencia‐Chingcuanco et al. 2007; Wan et al. 2008; Yang et al. 2017; Brinton et al. 2018).

As grain development progresses, genes associated with transport, proteolysis, carbohydrate metabolism and starch synthesis are upregulated, in addition to genes encoding storage proteins (Laudencia‐Chingcuanco et al. 2006; Pellny et al. 2012; Ma et al. 2014; Pfeifer et al. 2014; Yu et al. 2016). Interestingly, many studies have shown that genes with stress and defense‐related ontologies are also upregulated during grain development (Laudencia‐Chingcuanco et al. 2006; Nadaud et al. 2010; Capron et al. 2012; Ma et al. 2014; Brinton et al. 2018). Tissue‐specific expression studies also highlight the ongoing process of differentiation during grain development. For example, aleurone and endosperm tissues have similar gene expression profiles, at the early stages of grain development, but by later stages the expression profiles are very distinct (Gillies et al. 2012).

**Figure 3:**
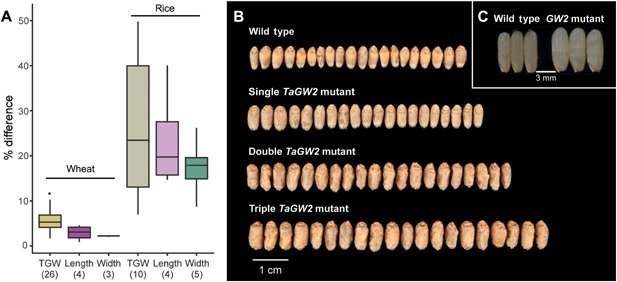
**Genetic control of grain weight in wheat** (**A**) The percentage effect of quantitative trait loci (QTL) for grain weight (measured as thousand grain weight; TGW), length and width in wheat and rice. The effect of single grain weight QTL in wheat is usually <10%, whereas in rice the effects are larger (mean = 25.9%). Values in brackets are the number of different QTL used to create the boxplots. Data used detailed in Table 1. (**B**) Effects of deleterious mutations in *TaGW2* (adapted from Wang et al. 2018b). Twenty representative grains are shown for each line. Combining mutations in multiple homoeologs has an additive effect on TGW compared to the wild type (single = 5.3%, double = 10.5%, triple = 20.7%) suggesting that there is functional redundancy amongst *TaGW2* homoeologs. (**C**) Effect of a natural loss of function mutation of *GW2* in rice (adapted from Song et al. 2007). This loss of function mutation results in both increased grain weight (49.8%) and grain width (26.2%) compared to the wild type.

Quantitative trait loci (QTL) for grain weight have been identified on almost every wheat chromosome (Huang et al. 2003; Breseghello and Sorrells 2007; Gegas et al. 2010; Kumar et al. 2016). However, only a subset of these QTLs have been validated and none have yet been cloned (Röder et al. 2008; Simmonds et al. 2014; Huang et al. 2015; Farré et al. 2016; Brinton et al. 2017). One of the major challenges to cloning grain size QTL in wheat is the subtle nature of the phenotypic effects, whereby single loci often increase average grain weight by <10%, and to an even lesser degree for the individual sub‐components (Figure 3A).

**Table 1:** Effects of grain weight and size QTL in Wheat and Rice

Species	Trait	Chromosome/QTL name	% difference	Reference
Wheat	TGW	2A	2.29	Farré et al. 2016
Wheat	TGW	5A	6.66	Farré et al. 2016
Wheat	TGW	6A	5.30	Farré et al. 2016
Wheat	TGW	5A	6.92	Brinton et al. 2017
Wheat	TGW	6A	3.96	Simmonds et al. 2014
Wheat	TGW	7D	10.00	Röder et al. 2008
Wheat	TGW	2D	6.80	Wu et al. 2014
Wheat	TGW	2D	10.30	Huang et al. 2003
Wheat	TGW	4D	11.70	Huang et al. 2003
Wheat	TGW	5B	4.80	Huang et al. 2003
Wheat	TGW	7A	3.80	Huang et al. 2003
Wheat	TGW	7B	4.90	Huang et al. 2003
Wheat	TGW	7B	5.80	Huang et al. 2003
Wheat	TGW	7D	6.60	Huang et al. 2003
Wheat	TGW	2D	4.25	Huang et al. 2015
Wheat	TGW	4B	4.45	Huang et al. 2015
Wheat	TGW	5A	5.50	Huang et al. 2015
Wheat	TGW	2B	7.16	Sukumaran et al. 2018
Wheat	TGW	2D	7.16	Sukumaran et al. 2018
Wheat	TGW	3B	4.50	Sukumaran et al. 2018
Wheat	TGW	5A	2.85	Sukumaran et al. 2018
Wheat	TGW	6A	1.70	Sukumaran et al. 2018
Wheat	TGW	7D	5.90	Sukumaran et al. 2018
Wheat	TGW	6A	3.71	Zhang et al. 2017b
Wheat	TGW	3D	7.23	Zhang et al. 2012
Wheat	TGW	2D	4.00	Breseghello and Sorrells 2007
Wheat	length	5A	2.14	Farré et al. 2016
Wheat	length	6A	0.85	Farré et al. 2016
Wheat	length	5A	4.04	Brinton et al. 2017
Wheat	length	7D	4.60	Röder et al. 2008
Wheat	width	5A	2.28	Farré et al. 2016
Wheat	width	6A	2.24	Farré et al. 2016
Wheat	width	6A	2.00	Simmonds et al. 2014
Rice	TGW	*TGW6*	10.00	Ishimaru 2003
Rice	TGW	*SRS3*	29.40	Kitagawa et al. 2010
Rice	TGW	*GS3*	46.29	Fan et al. 2006
Rice	TGW	*GW5*	22.90	Weng et al. 2008
Rice	TGW	*GL3*	43.50	Qi et al. 2012
Rice	TGW	*SW5*	12.80	Shomura et al. 2008
Rice	TGW	*GIF1*	24.00	Wang et al. 2008
Rice	TGW	*GW8*	13.90	Wang et al. 2012
Rice	TGW	*GS5*	6.98	Li et al. 2011b
Rice	TGW	*GW2*	49.80	Song et al. 2007
Rice	length	*SRS3*	14.70	Kitagawa et al. 2010
Rice	length	*GS3*	40.03	Fan et al. 2006
Rice	length	*GL3*	16.10	Qi et al. 2012
Rice	length	*SRS5*	23.40	Segami et al. 2012
Rice	width	*GW5*	19.60	Weng et al. 2008
Rice	width	*SW5*	17.90	Shomura et al. 2008
Rice	width	*GW8*	14.90	Wang et al. 2012
Rice	width	*GS5*	8.70	Li et al. 2011b
Rice	width	*GW2*	26.20	Song et al. 2007

TGW, thousand grain weight

This is exacerbated by the variation in grain weight that can exist within a single genotype, as discussed above, making unambiguous classification of individual lines as either high or low grain weight difficult. This ambiguity severely hampers genetic mapping, which relies on the clear separation between phenotypic categories. Some insight has been gained by breaking grain weight down into its individual morphometric sub‐components; e.g., grain length and width, which are under independent genetic control (Gegas et al. 2010).

Several studies in wheat have also identified associations for grain weight in naturally occurring variants of genes with demonstrated roles in model species, such as *Arabidopsis*. Many of these genes relate to starch metabolism, including ADP‐glucose pyrophosphorylase, sucrose synthase 2, and invertase genes (Jiang et al. 2011; Jiang et al. 2015; Hou et al. 2017), whereas other genes are predicted transcription factors (TFs; Zhang et al. 2015; Zhang et al. 2017a). While important as an initial step towards defining the commonality across species and for use in breeding, in most cases further functional validation would be warranted. This could be achieved using multiple independent mutants (Uauy et al. 2017), now available *in silico* (Krasileva et al. 2017), or gene‐edited or transgenic lines with the appropriate modification for each case.

This is important to conclusively show that the candidate genes and the proposed beneficial haplotypes are directly responsible for influencing grain weight, rather than being associated with increased grain size or weight, due to linkage disequilibrium with an unrelated nearby gene. This is especially relevant in species with uneven recombination across chromosomes, such as wheat and barley (Akhunov et al. 2003), as the decay in linkage disequilibrium could encompass dozens, if not hundreds, of genes.

An example of this is the centromeric region of chromosome 6A in wheat which has been associated with grain weight and yield in several studies and to which the wheat ortholog of rice *GRAIN WEIGHT 2* (*GW2*) gene has been mapped (Song et al. 2007; Simmonds et al. 2014; Sukumaran et al. 2015; Sukumaran et al. 2018; Zhang et al. 2018a). *TaGW2* has been validated as a negative regulator of grain size and weight in wheat through gene editing and mutant analysis (Yang et al. 2012; Simmonds et al. 2016; Wang et al. 2018b; Zhang et al. 2018b). However, care must be taken to associate haplotypes of *GW2* with these yield effects (Su et al. 2011; Zhang et al. 2013), given that linkage disequilibrium in this region is extensive. Sukumaran et al. (2018) reported a linkage block in this region between 77–81 cM, which encompasses 63% of the entire 6A chromosome (!) based on the physical wheat genome sequence (IWGSC 2018). Several studies have suggested the presence of alternative genes in the region influencing grain size and weight across environments (Simmonds et al. 2014; Avni et al. 2018; Sukumaran et al. 2018). Likewise, SNPs associated with increased grain weight in one panel, are not always validated in an independent association panel (e.g., TaGS5; Ma et al. 2015; Zhang et al. 2018a).

Despite the limitations outlined above, studies in model species do provide a valuable starting point for the functional characterization of genes and understanding their effect on grain weight in wheat. In rice, over 400 grain weight QTL have been identified, and several of the underlying genes have been cloned (reviewed in Xing and Zhang 2010; Huang et al. 2013). Studies in *Arabidopsis* have also provided a deep molecular insight into the control of seed size (reviewed in Li and Li 2015, 2016).

The gene underlying the rice *GW2* QTL (Song et al. 2007) mentioned previously provides a good case study of how knowledge from model species is being translated into wheat and used to inform our understanding of grain weight. *GW2* is an E3 ubiquitin ligase that negatively regulates grain width, and this mechanism appears to be conserved across species, including wheat, *Arabidopsis* and maize, on both a cellular and molecular level (Li et al. 2010; Xia et al. 2013; Simmonds et al. 2016). The wheat ortholog, *TaGW2*, was shown to affect cell number in maternal tissue and to have ubiquitination activity, as in rice and *Arabidopsis* (Bednarek et al. 2012; Xia et al. 2013; Dong et al. 2017; Zhang et al. 2018b).

Another example is *KLUH*, an *Arabidopsis* cytochrome P450 shown to positively regulate seed size through the promotion of cell proliferation in the integuments (Adamski et al. 2009). Virus induced gene silencing (VIGs) in the grain of a wheat gene from the same cytochrome family, *TaCYP78A5*, resulted in a reduction in grain size of 11%, due to reduced cell proliferation in the ovary and developing seed, suggesting a conserved mechanism (Ma et al. 2016).

Caution should be exercised, however, when transferring knowledge between species, as there are fundamental differences in seed development and, therefore, not all gene functions will be conserved. For example, heterotrimeric G‐protein complexes consist of three subunits: Gα, Gâ and Gγ and roles in seed/grain size regulation have been identified for examples of all subunits in *Arabidopsis* and rice (reviewed in Botella 2012). However, it is not clear if function is completely conserved across species. For example, an *Arabidopsis* Gα subunit, *AGG3*, positively regulates seed size, while the most similar rice Gα subunits*, DEP1* and *GS3* appear to be negative regulators of seed size (Fan et al. 2006; Huang et al. 2009; Li et al. 2012). It is not yet clear what role this pathway plays in regulating grain weight in wheat.

One major difference between the grain weight QTL identified in species such as rice, compared to those identified in wheat, is the magnitude of the effects. As mentioned previously, the average effect of a single locus in wheat is usually <10%, whereas major grain weight QTL in rice often increase grain weight by >20% (Figure 3A). The same is true in *Arabidopsis*, with the knockout of single genes routinely modulating seed size by approximately 20% (Garcia et al. 2005; Adamski et al. 2009; Xia et al. 2013).

It has been proposed that the subtlety of these effects in wheat is due to its polyploid nature, compared to the diploid status of rice and *Arabidopsis* (Borrill et al. 2015). Bread wheat is a hexaploid, consisting of three homoeologous genomes (A, B and D) and, as such, most genes exist as three closely‐related copies sharing ≥95% sequence similarity. Functional redundancy between homoeologs can, therefore, result in the effects of variation in a single gene being masked, completely or in part, by the effects of the remaining functional copies. Indeed, variation in the *GW2* gene in rice leads to grain weight differences of up to 50%, whereas a similar mutant in a single genome of the wheat ortholog (*TaGW2‐A*) affects grain weight by only approximately 7% in wheat (Song et al. 2007; Simmonds et al. 2016). This hypothesis is supported by the fact that downregulating multiple homoeologs of *TaGW2* has an additive effect, with simultaneous downregulation of all three homoeologs, by gene editing and mutants, increasing grain weight by 16.3% and 20.7%, respectively (Figure 3B; Wang et al. 2018b).

## MECHANISTIC CONTROL OF GRAIN WEIGHT

In addition to understanding the genetic basis of grain weight, determining the mechanism by which these genes work is critical to inform strategies for yield improvement. Studies in model species have revealed that seed size is controlled by genes with a diverse range of molecular functions. TFs belonging to many different families have been shown to be involved in the control of seed size; e.g., the rice *SQUAMOSA PROMOTER‐BINDING LIKE* (*SPL*), *OsSPL16*. This TF was cloned as the gene underlying the rice *GRAIN WIDTH 8* (*GW8*) QTL and positively regulates grain size (Wang et al. 2012).

Genes involved in the ubiquitin pathway are also important regulators of grain weight in many plant species, including *GW2* (reviewed in Li and Li 2014). Genes with deubiquitinating activity have also been shown to influence seed size, such as *WIDE AND THICK GRAIN 1* (*WTG1*) in rice and *UBIQUITIN SPECIFIC PROTEASE 15* (*UBP15*) in *Arabidopsis*, which act as negative and positive regulators of seed size, respectively (Du et al. 2014; Huang et al. 2017). Genes involved with phytohormone signaling are also important regulators of seed size, with roles being demonstrated for auxin, brassinosteroid and cytokinin biosynthesis and signaling components (Riefler et al. 2006; Schruff et al. 2006; Jiang et al. 2013).

Components of many other pathways have also been shown to influence grain and seed size in multiple species, including the heterotrimeric G‐protein signaling pathway (discussed above; Botella 2012), MAP‐kinase cascades (Xu et al. 2018), epigenetic factors (Xiao et al. 2006) and sugar metabolism (Ohto et al. 2005; Ohto et al. 2009). Here, we discuss some of the mechanisms through which seed size can be regulated, both on a molecular level considering the mechanistic action of specific genes and on a whole organ level. Where possible we refer to studies in wheat, but also draw from work performed in model species such as rice and *Arabidopsis*.

## CELL SIZE vs CELL NUMBER

Grain weight and its individual morphometric parameters are determined by the modulation of cell size and cell number. A range of genes with different molecular functions influence seed size through either the positive or negative regulation of cell number. *GW2* exerts its control over seed size through limiting cell division in rice, wheat and *Arabidopsis* (Song et al. 2007; Xia et al. 2013; Zhang et al. 2018b). *DA1*, a downstream target of the *Arabidopsis GW2* ortholog, also negatively regulates cell number and the two genes work to influence seed size in a synergistic manner (Xia et al. 2013; Dong et al. 2017). On the other hand, *OsSPL16* positively regulates grain size through the promotion of cell proliferation (Wang et al. 2012).

Cell size in the seed can be modulated either directly or indirectly. Some genes act to physically modify the cell wall and alter its properties (reviewed in Cosgrove 2005). Expansins and *XTH* genes disrupt cross links in the cell wall, allowing for increased turgor driven cell expansion, and the expression of these genes has been associated with cell and grain length in wheat and barley (Lizana et al. 2010; Radchuk et al. 2011; Munoz and Calderini 2015). In *Arabidopsis*, the WRKY TF *TTG2* regulates some steps of the tannin biosynthesis pathway. *ttg2* mutants have smaller seeds due to smaller cells in the seed coat, likely due to altered tannin levels in the cell wall, resulting in a reduced capacity for elongation (Johnson et al. 2002; Garcia et al. 2005). Microtubule dynamics are also important determinants of cell size and shape (Li et al. 2011a; Fujikura et al. 2014). The rice grain weight gene *SRS3* was shown to be a kinesin 13 protein, a family of genes which regulate microtubule depolymerization (Kitagawa et al. 2010).

Other genes regulate cell size in the seed through more indirect mechanisms, for example, through the regulation of sugar metabolism and subsequent accumulation in the vacuole, and endoreduplication (Ohto et al. 2005; Ohto et al. 2009; Chevalier et al. 2014). We recently showed that a grain weight QTL, on wheat chromosome 5A, is associated with increased grain length due to increased cell size in the pericarp. Whether this is a direct or indirect effect remains to be determined (Brinton et al. 2017).

## MATERNAL CONTROL OF GRAIN SIZE

Many of the genes shown to regulate seed size in rice and *Arabidopsis* appear to act maternally, either pre‐ or post‐fertilization (reviewed by Li and Li 2015). A strong association between carpel size and final grain weight has been documented in wheat (Calderini et al. 1999; Calderini and Reynolds 2000; Hasan et al. 2011; Xie et al. 2015; Reale et al. 2017). *TaGW2* acts maternally, with 10% increased carpel size in the A‐genome knock‐out mutant lines compared with wild‐type (Simmonds et al. 2016). The rice and *Arabidopsis* ortholog of *GW2* also act maternally to modulate seed size (Song et al. 2007; Xia et al. 2013).

The mechanisms by which the carpel and its component tissues influence final grain size are not well understood in wheat and barley. Reale et al. (2017) established that the size of both the ovary wall and the ovule itself were associated with larger carpels and grains, largely due to cell number, rather than size. However, the two tissues were not affected to the same extent, resulting in an increased ovule:ovary wall ratio in larger grains. The ovule and its component tissues play an important role during fertilization and subsequent grain development (reviewed in Wilkinson et al. 2018). Variation in nucellus size has been observed between barley cultivars but if and how this influences final grain size remains to be determined (Wilkinson and Tucker 2017).

Maternally acting genes may not necessarily increase carpel size, but instead, may affect tissues of maternal origin, later during grain development. For example, the sucrose transporters *SUT1* and *SUT2* are highly expressed in nucellar tissue and the nucellar projection in the days immediately following fertilization, and downregulation of these genes in barley has been associated with decreased grain weight (Radchuk et al. 2017). PCD in the pericarp tissue, arising from the maternal ovary wall has also been shown to be important for the maternal control of grain size. Downregulation of *VACUOLAR PROCESSING ENZYME 4* (*VPE4*) by RNAi in barley resulted in delayed PCD in the pericarp and consequently smaller grains (Radchuk et al. 2018). PCD is thought to be an important step for enlargement of the pericarp to accommodate endosperm growth (Radchuk et al. 2011; Radchuk et al. 2018), highlighting the fact that grain development involves the tight coordination of processes across multiple tissues, which will ultimately determine the final size of the grain.

In multiple species, including wheat, it has been proposed that the size of the maternal outer layers determines the final grain size by placing a physical upper limit on the space into which the endosperm can grow (Calderini et al. 1999; Adamski et al. 2009; Hasan et al. 2011). Studies in wheat showing a correlation between carpel size and dry matter accumulation support this hypothesis, suggesting that increasing the size of the maternal tissues allows an enhanced capacity for grain filling i.e. increased source strength (Calderini and Reynolds 2000; Xie et al. 2015; Brinton et al. 2017). The maternal parent will also contribute to final seed size through other mechanisms including responses to the environment during grain development and the imprinting of genes after fertilization, both of which have been shown to influence final grain size (discussed in Zhang et al. 2016a).

Studies in *Arabidopsis* suggest that increased size of the maternal seed coat can be achieved as an indirect effect of increased growth of the endosperm, a zygotic tissue. For example, the *HAIKU* (*IKU*) genes act to promote endosperm growth in *Arabidopsis*. The *iku* mutants have smaller seeds, due to reduced endosperm growth and, indirectly, reduce cell elongation in the seed coat (Garcia et al. 2003). The indirect effect on cell size, in the seed coat, was determined by demonstrating that *iku* double mutants pollinated with WT pollen had WT‐like seeds, therefore showing that the *iku* mutations do not have a direct effect on the maternal seed coat. Already this suggests a level of communication between the endosperm and seed coat, extending beyond purely mechanical forces (Garcia et al. 2003).

The *IKU* genes interact on a genetic basis with *TTG2* and *iku ttg2* double mutants have seeds even smaller than *iku* mutants, due to the *ttg2* mutation compromising the elongation capacity of the cell walls in the seed coat, and hence, further restricting endosperm growth. This is in accordance with the size of the maternal pericarp imposing a physical constraint on endosperm growth. However, combining the *iku* mutations with lines that have reduced cell proliferation in the seed coat (due to overexpression of *KIP RELATED PROTEIN2*) does not have an additive effect on seed size, and instead, the reduction in cell number in the seed coat is compensated for by increased cell elongation (Garcia et al. 2005). This suggests that, in some cases, the size of the seed coat can be adjusted to accommodate the growth of the endosperm, providing additional evidence there must be communication between the tissues.

This communication is also present in wheat, as exemplified for seed dormancy, where the *R* genes, which determine grain color specifically in the seed coat, have pleiotropic effects on embryo dormancy (Flintham 2000; Himi and Noda 2005). Studies in maize have shown that the maternal outers layers are important for specifying the fate of some, but not all, cell types in the endosperm. *In vitro* cultures of maize endosperm develop aleurone cells on the surface, in the absence of maternal layers, however, differentiation of transfer cells does not occur. Interestingly, the *in vitro* grown endosperms grew for roughly the same duration as those grown *in planta*, and underwent similar frequencies of cell divisions, suggesting that the growth duration of the endosperm is somewhat independently controlled (Gruis et al. 2006).

The exact nature of the communication between tissues is not fully understood. In *Arabidopsis*, roles have been demonstrated for phytohormones, epigenetic factors and sugars (Nowack et al. 2010; Locascio et al. 2014; Radchuk and Borisjuk 2014) but relatively little is understood about the molecular basis of this signaling in cereals.

Distinguishing between true maternal effects and zygotic effects that, indirectly, influence maternal tissue cannot always be achieved by phenotypic characterization, particularly when considering post‐anthesis effects. For example, the grain weight QTL on wheat chromosome 5A increases grain length due to longer cells in the pericarp tissue. However, the QTL does not influence carpel size and the first differences in grain size are only observed several days post‐fertilization (Brinton et al. 2017). In these cases, assigning the effect as maternal, or zygotic, requires experiments involving reciprocal crosses and phenotypic assessment in F_1_ hybrid seed.

Experiments of this nature in wheat, however, are challenging as the subtle effects of grain weight genes (Figure 3A) are likely to be masked by the phenotypic variation observed across F_1_ grains. Understanding the precise mechanism by which the grain weight is being controlled can help to make these experiments possible. In the case of the 5A grain weight QTL, we showed that the effect on pericarp cell size was independent of individual grain size, suggesting that this more robust phenotype could be used to assess the F_1_ grains (Brinton et al. 2017).

## PHYSICAL CONSTRAINTS TO GRAIN SIZE

As discussed above, the interaction of different tissues *within* the grain imposes mechanical forces and physical constraints that determine the final size of the grain. In addition, there is also evidence that the size of the grain can be influenced by physical constraints imposed by other, non‐grain tissues, such as the lemma and palea, which define the floret cavity size (Figure 1E). In rice, for example, the palea and lemma form a tight‐fitting enclosure, the spikelet hull, that restricts the growth of the grain and defines the potential for the final grain size, even before the grain has formed (Lombardo and Yoshida 2015).

In wheat, the lemma and palea envelop the grain as in rice, but can separate and allow the grain to grow further out of these structures. Studies have shown, however, that the physical pressure exerted by the glume, palea and lemma inhibit grain expansion in wheat. This leads to high correlations (mean *r* = 0.65) between the floret cavity size, that is defined by these floral structures, and final grain weight (Millet and Pinthus 1984; Millet 1986). Natural variation for glume, lemma, and palea size has been described in wheat, most famously in *Triticum polonicum*, a tetraploid sub‐species of wheat that has long glumes and long grains and was first documented by the Swedish botanist Linnaeus in the 18^th^ century (Percival 1921).

The long glume phenotype has been mapped as a single semi‐dominant locus (dubbed *P1*) on chromosome 7A, and studies confirmed the linkage between glume length and grain size conferred by the *P1* locus (Watanabe et al. 1996; Okamoto and Takumi 2013). Given that the floral structures that define floret cavity size are borne from the mother plant, this exemplifies yet another mechanism of maternal control of grain size in cereals.

## INTERACTIONS

Final grain size and weight are defined by a series of complex interactions which are integrated across the life cycle of the crop. These extend beyond the traditionally defined compensatory effects between the major yield components (spike/m^2^; grain number/spike; grain weight) and include interactions within each of these components. For example, grain weight results from the integration of sub‐components, such as cell division, cell expansion and grain filling rate, among other processes. Moreover, these interactions play out within an environmental context of weather patterns, biotic stress and field management practices across each growing season, which affects these relationships in different ways and to differing degrees. Therefore, when measuring final grain size and weight, we are measuring the integration of these complex and dynamic events across the life cycle.

We argue that a more detailed understanding of how particular genes and QTL affect individual yield components will allow a more nuanced understanding of these interactions. For example, traditionally grain number and grain size have been considered to be negatively correlated, due to competition for assimilates during grain filling (Sadras 2007; Guo et al. 2016). However, some genes (e.g. *GW2*) exert their control on grain weight by affecting carpel size, a yield component that is determined during the same growth stages as the definition of grain number (Simmonds et al. 2016; Reale et al. 2017). Hence, for these cases, the interactions between grain number and grain size could be due to competition for resources pre‐anthesis, and not necessarily due to any post‐anthesis events relating to grain filling. Therefore, being able to break down yield components into their constitutive traits, in this example carpel size, due to cell division as opposed to the more general final grain weight, should allow a more in‐depth and meaningful characterization of these interactions and potential trade‐offs. This will help define the relationships between individual factors more clearly and avoid grouping multiple aspects of yield into a single component.

The more precise definition of how individual genes and QTL affect specific yield components will also allow a more rational and targeted combination of traits. This is now becoming increasingly possible as we further define QTL and identify the genes and haplotypes that underlie natural variation for many of these yield components. In addition, knowledge from model species is becoming increasingly simpler to transfer into wheat, using either sequenced mutant populations or gene editing (Zhang et al. 2016b; Krasileva et al. 2017). This knowledge will allow the combination of genes which affect distinct processes governing grain size (e.g. cell division and cell expansion), through different mechanisms and pathways (e.g. ubiquitin and phytohormones), as well as at distinct developmental stages (e.g. carpel development and grain filling rate).

We expect that these combinations will allow more in‐depth study of potential compensatory effects between yield components than has been possible before, and bypass possible epistatic interactions when genes affecting the same developmental processes or molecular mechanisms are combined. We also hypothesize that this strategy of combining complementary mechanisms will provide a more resilient genetic basis for yield stability, under changing weather patterns, as positive alleles will be operating during different growth stages and through various molecular pathways.

Recent examples in rice showcase this approach, where combining mutations in two negative regulators of grain size (GS3; Fan et al. 2006) and grain number (GN1a; Ashikari et al. 2005) increased grain size and number across all ten genotypes tested (Shen et al. 2016). However, these positive effects only translated into yield increases for three of the ten cultivars. In seven cultivars, tillering was reduced in the double mutants, leading to a significant reduction in yield (Shen et al. 2016). This highlights the importance of testing these combinations in multiple genetic backgrounds as compensatory effects will most likely differ, as exemplified in the rice *gs3/gn1a* double mutant.

To date many of the interactions between individual genes have been conducted using near‐isogenic lines which differ for the specific genes being studied (Gao et al. 2015). This requires multiple rounds of backcrossing to combine the traits, which, despite accelerated growth conditions (Watson et al. 2018), is cumbersome and time consuming. In the future, understanding the relationship between multiple genes affecting yield components will most likely require multiplex genome editing of the different targets across several genetic backgrounds (Shen et al. 2017). This will involve not only knock‐out mutations but also allele replacement and promoter manipulations (Puchta 2017; Rodríguez‐Leal et al. 2017; Li et al. 2018; Ran et al. 2018).

This targeted editing approach will also be complemented by empirical yield data stemming from breeding programs. Here, large populations segregating for different alleles and haplotypes, across the genes of interest, can be analyzed retrospectively (Bevan et al. 2017) to determine which combinations have succeeded within different breeding programs and environments, potentially identifying additional genes that allow specific allelic combinations to manifest into on‐farm yield. A complementary approach to identifying beneficial alleles in existing cultivars will be to explore landraces and wild progenitor species, such as tetraploid wild emmer (*T. dicoccoides*) and diploid goatgrass (*Aegilops tauschii*), as sources for novel genes and variation associated with grain weight.

Recent genetic studies have shown the potential for these wild species to provide useful genetic variation for grain weight that could possibly have been excluded from the gene pool during domestication (Golan et al. 2015; Arora et al. 2017; Avni et al. 2018). The genome sequences and other genomic resources now available for many of these progenitor species and landraces will aid the identification of these novel genes and alleles (Avni et al. 2017; Luo et al. 2017; Wingen et al. 2017).

In addition to combining effects between different mechanisms and developmental timings, the cloning of genes will allow the combinations of beneficial alleles across homoeologs. This will be important due to the subtle effects of grain weight QTL in hexaploid wheat compared to grain weight QTL in diploid species (Figure 3A). Simultaneously modulating the function of all three homoeologs has the potential to expand the range of phenotypic variation and achieve effects comparable to those in diploids, as evidenced by the *GW2* example (Figure 3B) and additional traits (Song et al. 2007; Borrill et al. 2015; Wang et al. 2018b).

These allelic combinations should provide breeders with novel and extended phenotypic variation as beneficial mutations in all three homoeologs are unlikely to occur simultaneously in natural populations (Uauy 2017; Uauy et al. 2017). The larger phenotypic effects expected from recessive mutations might help break some of the negative compensatory effects often observed with the subtle single locus effects. This extended variation might also prove important for understanding gene function. To date, it has been difficult to compare physiological traits in isogenic lines with significant, yet subtle effects, on yield components.

In many cases, the phenotypic variation in secondary traits is even smaller than the primary effect, which means that often these effects cannot be separated from the inherent variation observed in field trials. This makes it extremely difficult to establish causal relationships between genes affecting specific yield components and compensatory effects. The stronger phenotypic effects of double or triple mutants should amplify the phenotypic signal making it easier to establish causality by distinguishing ‘true’ effects from inherent background variation.

Grain size and weight are affected by events occurring both pre‐anthesis (e.g. the definition of grain number, carpel size, stem water soluble carbohydrate reserves), and post‐anthesis (e.g. grain cell elongation, grain filling rate). While grain filling is most commonly considered to be sink‐limited, events pre‐anthesis are most commonly considered to be source‐limited (Slafer and Savin 1994; Borrás et al. 2004; Slafer et al. 2005; Miralles and Slafer 2007; González et al. 2014). Therefore, this suggests that it is important to consider interactions between source and sink tissues and understand how they can be simultaneously improved. Recent advances in understanding of photosynthesis and the effects of light/shade transitions promise to improve biomass accumulation due to increase CO_2_ assimilation (Driever et al. 2017; Taylor and Long 2017). Empirical selection for high biomass lines is also helping improve yield potential in breeding programs (Reynolds et al. 2017).

Non‐genetic approaches have also been used to manipulate source‐sink relationships. Griffiths and colleagues recently manipulated T6P levels in wheat, post‐anthesis, using chemical applications of T6P signaling‐precursors (Griffiths et al. 2016). They show that application of the precursors, at 10 dpa, significantly increased grain size in wheat. However, no pre‐anthesis applications were reported. Given that T6P affects both source and sink tissues (Figueroa and Lunn 2016), it will be of interest to determine the effect of modulating this signaling metabolite, at earlier growth stages, to determine if this impacts on additional yield components.

Likewise, CO_2_ enrichment studies have shown increases in wheat yield due to increased tillering, grain number and grain size (Wang et al. 2013). The positive effect, however, may be tempered by the fact that the rate of CO_2_ assimilation may exceed potential sink capacity of the grains. For future CO_2_ enrichment and T6P precursor studies it would be extremely informative to use the germplasm described above which combines different and potentially complementary yield component traits, as well as double or triple mutants.

## CHALLENGES AND OPPORTUNITIES

A fully annotated wheat genome is now available (IWGSC 2018) providing new opportunities to identify genes controlling grain size and weight. If the advances in the rice community are used as a comparison, we should expect a significant increase in the number of genes being cloned and characterized in wheat. This enthusiasm is tempered by the subtle phenotypes observed in wheat, compared to diploid species, and the inherent difficulties of working in the polyploid system. However, the combination of tools and resources being generated, such as expression atlases and networks (IWGSC 2018; Ramírez‐González et al. 2018), sequenced mutants (Krasileva et al. 2017) and gene‐editing technologies (Zhang et al. 2016b), should position the community in the best possible place to start understanding the genes and pathways that control grain size and weight.

The identification of genes controlling these traits will allow novel combinations to be established, both in terms of variation across homoeologs, as well as combinations of distinct mechanisms that affect grain size in different ways. This germplasm should extend current phenotypic variation and will facilitate more detailed physiological analyses of potential trade‐offs between the multiple grain yield components. Likewise, new and improved phenotyping technologies, such as high‐throughput profiling of grains across individual florets and spikelets to detailed bioimaging and topological growth maps (Pielot et al. 2015), will help take full advantage of these genetic stocks to advance our understanding of the processes governing yield components.

The ability to re‐sequence cultivars, as already done in rice (Wang et al. 2018a), will also soon allow researchers and breeders to leverage multiple years of available data to better understand existing variation. Integrating and modelling this physiological, mechanistic and genomic information should aid in defining breeding priorities and determining which novel alleles should be engineered, introduced, and combined. This will allow us to move beyond the reductionist approach towards a more integrated framework based on understanding of the constituent parts. The challenge remains, however, to ensure that this knowledge is deployed into cultivars and that it delivers in farmers’ fields to maximize yield and stability under real‐world agronomic practices. We will be judged by our success in ensuring that this happens.

## AUTHOR CONTRIBUTIONS

J.B. and C.U. conceived and wrote the review, and all authors read and approved the contents of this paper.
